# Synthesis of a dihalogenated pyridinyl silicon rhodamine for mitochondrial imaging by a halogen dance rearrangement

**DOI:** 10.3762/bjoc.15.226

**Published:** 2019-10-01

**Authors:** Jessica Matthias, Thines Kanagasundaram, Klaus Kopka, Carsten S Kramer

**Affiliations:** 1 Max Planck Institute for Medical Research, Department of Optical Nanoscopy, Jahnstraße 29, 69120 Heidelberg, Germany; 2 Helmholtz International Graduate School, German Cancer Research Center (DKFZ), Im Neuenheimer Feld 223, 69120 Heidelberg, Germany; 3 Division of Radiopharmaceutical Chemistry, German Cancer Research Center (DKFZ), Im Neuenheimer Feld 223, 69120 Heidelberg, Germany; 4 Institute of Inorganic Chemistry, Im Neuenheimer Feld 270, 69120 Heidelberg, Germany; 5 German Cancer Consortium (DKTK), Heidelberg, Germany

**Keywords:** halogen-dance reaction, mitochondrial probe, near-infrared (NIR) dyes, one-pot reaction, silicon rhodamines

## Abstract

**Background:** Since their first synthesis, silicon xanthenes and the subsequently developed silicon rhodamines (SiR) gained a lot of attention as attractive fluorescence dyes offering a broad field of application. We aimed for the synthesis of a fluorinable pyridinyl silicon rhodamine for the use in multimodal (PET/OI) medical imaging of mitochondria in cancerous cells.

**Results:** A dihalogenated fluorinatable pyridinyl rhodamine could be successfully synthesized with the high yield of 85% by application of a halogen dance (HD) rearrangement. The near-infrared dye shows a quantum yield of 0.34, comparable to other organelle targeting SiR derivatives and absorbs at 665 nm (ε_max_ = 34 000 M^−1^cm^−1^) and emits at 681 nm (τ = 1.9 ns). Using colocalization experiments with MitoTracker^®^ Green FM, we could prove the intrinsic targeting ability to mitochondria in two human cell lines (Pearson coefficient >0.8). The dye is suitable for live cell STED nanoscopy imaging and shows a nontoxic profile which makes it an appropriate candidate for medical imaging.

**Conclusions:** We present a biocompatible, nontoxic, small molecule near-infrared dye with the option of subsequent radiolabelling and excellent optical properties for medical and bioimaging. As a compound with intrinsic mitochondria targeting ability, the radiolabelled analogue can be applied in multimodal (PET/OI) imaging of mitochondria for diagnostic and therapeutic use in, e.g., cancer patients.

## Introduction

Since their first synthesis by Fu and co-workers in 2008 [1], silicon xanthenes and the subsequently developed silicon rhodamines (SiR) have drawn a lot of attention as attractive fluorescence dyes offering a broad field of application. Their excellent spectral (absorption and emission bands in the near-infrared region), photophysical (bright and photostable) and biochemical (biocompatible, biological stable and cell membrane permeable) properties make them useful tools in live cell super-resolution microscopy [2–9], as direct probes for various biomolecules [10–13] or as sensors for metal ions [14–18], pH [16], voltage [19] or metabolites [20–23]. Several attempts were made, partially supported by DFT calculations, to correlate the dyes’ structural features with their optical properties and control the latter by rational dye design [15,24–26]. These investigations led to new silicon rhodamine dyes with enhanced and fine-tuned properties (quantum yield, lifetime, brightness, absorption and emission maxima). A recent review compared the photophysical properties of numerous silicon rhodamines leading to further insights into the correlation of the dyes’ chemical structure with their fluorogenic behavior [27]. Regarding the quantum yield, Hanaoka et al. have shown that introduction of methyl, methoxy or dimethylamine groups into the benzene moiety of silicon rhodamines could tune the HOMO energy level [15]. Depending on the oxidation potential and the HOMO energy level of the benzene moiety, the quantum yield was greatly altered but absorption and emission bands remained unchanged. Thus, the quantum yield shows a direct connection to the negative value of the HOMO energy level and/or the oxidation potential [15], but it is also influenced by other factors. Nonradiative quenching (e.g., bond rotation) can effectively contribute to depopulation of the fluorophore’s excited state [28], yielding a lower quantum yield. However, the rational trends for both radiative and nonradiative decays still remain difficult to predict despite theoretical and experimental efforts of the past years [29]. Here we initially assume that, in analogy to BODIPY fluorophores [30], restricted rotation around the xanthene aryl bond should lead to an improved quantum yield.

**Scheme 1:**
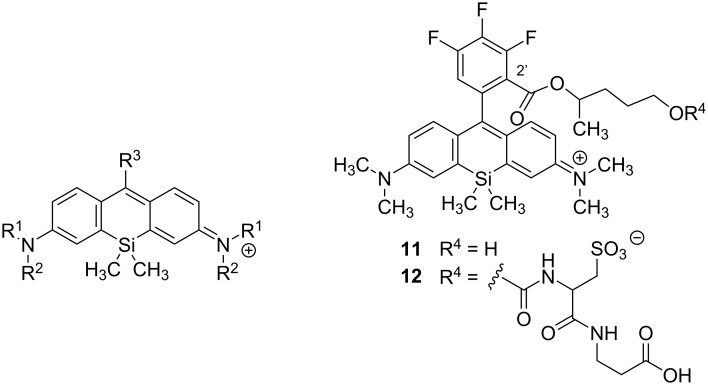
Comparison of optical properties of different silicon rhodamines.

In Scheme 1 we compile silicon rhodamines with high quantum yields as well as their structural analogues with lower quantum yields. Silicon fluoresceins were excluded from this compilation, although “2-COOH DCTM” [31], “2-COOH DFTM” [31], and “Maryland red” [32] are representatives with quantum yields up to 0.67. As described previously, azetidine substituents at the xanthene moiety of **5**, **7**, and **9** lead to an improved quantum yield and to a red shift in comparison to the *N*,*N*-dimethylaniline analogues **1**, **3**, and **4** (Table 1, entry 1 vs 5, 3 vs 7, and 4 vs 9). In contrast, the 4-fluoroazetidine moiety in **10** (“JF_635_”) causes a hypsochromic shift without affecting the high quantum yield compared to the azetidine analogue **9** (“JF_646_”) (Table 1, entry 9 vs 10). Comparing the phenyl substituted rhodamine **2** with its 2’-methyl substituted analogue **3**, restricted rotation around the xanthene–benzene bond leads to a drastic improvement of the quantum yield from 0.10 to 0.31 (Table 1, entry 2 vs 3). Accordingly, rhodamines like **3**, **4**, **11**, or **12** bearing 2’-substituents with A-values between a proton and a methyl group (such as F and Cl) show quantum yields from 0.19 (for F) [23] to 0.30 (for Cl) [23]. This observation leads us to the hypothesis that the quantum yield correlates positively with the bulkiness of the phenylic 2’-substituent, which restricts the rotation around the xanthene–benzene bond more strongly the larger it is.

**Table 1:** Comparison of optical properties of different silicon rhodamines.

Entry	NR^1^R^2^	R^3^		λ_abs_	λ_em_	Φ	Ref

1	N(CH_3_)_2_	H	**1** (SiP)	634	648	0.42^a,b^	[15]
2	N(CH_3_)_2_	Ph	**2**	646	667	0.10^b,c^	[33]
3	N(CH_3_)_2_	2-CH_3_-Ph	**3**	646	660	0.31^a,b^	[15]
4	N(CH_3_)_2_	2-COOH-Ph	**4**	643	662	0.41^d,e^	[24]
5	azetidine	H	**5**	636	649	0.62^d,e^	[32]
6	azetidine	COOH	**6**	641	657	0.26^d,e^	[32]
7	azetidine	2-CH_3_-Ph	**7**	649	663	0.47^d,e^	[32]
8	azetidine	2-CH_3_-6-CH_3_-Ph	**8**	651	664	0.51^d,e^	[32]
9	azetidine	2-COOH-Ph	**9** (JF_646_)	646	664	0.54^d,e^	[24]
10	3-fluoroazetidine	2-COOH-Ph	**10** (JF_635_)	635	652	0.56^d,e^	[25]
11			**11**	662	680	0.66^f,g,h^	[34]
12			**12**	663	680	0.70^f,h^	[34]
13	N(CH_3_)_2_	pyridin-4-yl	**13**	655	680	0.12^c,e^	[35]
14	azetidine	3-methylpyridin-4-yl	**14**	656	670	0.48^d,e^	

^a^In PBS buffer at pH 7.4; ^b^cresyl violet in methanol was used as reference dye; ^c^in PBS buffer; ^d^in HEPES buffer at pH 7.3; ^e^quantum yield was determined by absolute measurement; ^f^in water; ^g^the corresponding isopropyl ester showed a quantum yield of 0.60; ^h^quantum yield determined in water with the reference dye Atto AZ 237.

Remarkably, dyes **1**, **5** and **6** already possess moderate to high quantum yields without any benzene moiety (Table 1, entries 1, 5 and 6). The fluorophores **11** and **12** exhibit values of 0.66 and 0.70 and have, to the best of our knowledge, the highest quantum yields known amongst silicon rhodamines (Scheme 1, Table 1, entries 11 and 12). One reason might be the bulkiness of the ester group in 2’-position, an isopropyl ester derivative; another one might be the effects of the fluorine atoms and the ester group on the HOMO energy level of the benzene moiety.

**Scheme 2:**
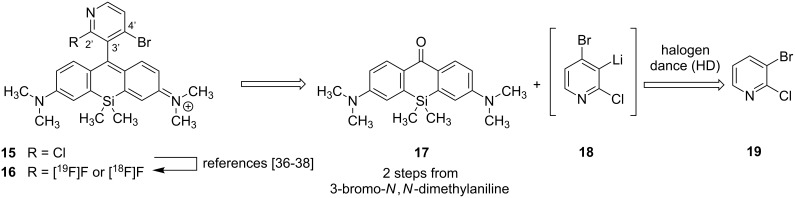
Retrosynthetic analysis of the proposed small molecule bimodal probe [^18^F]**16** for both optical and PET imaging of cancer cells with up-regulated mitochondrial activity.

Since our group is interested in PET-active near-infrared (NIR) dyes for bimodal medical imaging (PET/optical imaging (OI)), we wanted to develop the pyridinyl-substituted silicon rhodamine **15**, in which the 2-halopyridine moiety can easily be radiofluorinated to the PET-active dye **16** (Scheme 2, for examples on radiolabelling of 2-halopyridines see [36–38] and references therein). While the PET modality is highly interesting for precise medical imaging of diseases, the fluorescence modality can be utilized for medical interventions, such as fluorescence-guided surgery or sentinel lymph node detection or for histopathological analyses of biopsy material.

To date, only two pyridinyl-substituted silicon rhodamines have been published. Dye **13** (Scheme 1, Table 1, entry 13) shows good water solubility, has a quantum yield of 0.12 and offers intrinsic targeting ability to lysosomes [35]. Pyridine silicon rhodamine **14** (Table 1, entry 14) has an improved quantum yield of 0.48 [32], presumably due to the restricted rotation of the xanthene pyridine bond.

The 2-chloropyridinyl moiety in **15** is not only attractive because of its option for convenient fluorination, but also since its intrinsic targeting ability to acidic cellular compartments (such as the lysosomal-selective fluorophore **13**). We assumed that our proposed bimodal fluorophore would show a better selectivity for mitochondria because of its more lipophilic nature (clogP for **15** 3.712, for **16** 3.142) compared to **13** (clogP 2.136). Medical imaging of mitochondrial activity is highly interesting for various indications, ranging from neurodegenerative and metabolic diseases to ischemic injuries, necrosis, therapy response and cancer [39–43]. Because many cancer cells have a higher mitochondrial membrane potential than nontransformed cells [41,44–45], we believe that our imaging agent will achieve a reasonable tumor-to-background ratio.

Lipophilic cations such as the phosphonium cation or rhodamines are known to accumulate selectively within the mitochondria, driven by the mitochondrial plasma membrane potential [39–40]. Thereby, the high lipophilicity facilitates the diffusion through the lipid bilayers of the cell and mitochondrial membranes. Recently, a silicon rhodamine for selective mitochondrial staining was developed by conjugation of the SiR core with ten different amines varying in lipophilicity [46]. The authors showed that the optimal range of clogP values for mitochondrial targeting ranges from 5.50 to 6.33.

As mentioned above, the pyridinyl-substituted silicon rhodamines **13** and **14** are dyes with spectral properties in the near-infrared region. Dye **14** possess the higher quantum yield not only due to the azetidine substituents at the xanthene moiety, but also because of the restricted bond rotation owing to the 3’-methyl (pyridine numbering) group. Since the smaller 2’-fluorine substituent in **16** should lead to a decrease in quantum yield compared to **15**, we aimed for a molecule with an additional bulky 4’-substituent such as bromine. The bromine should not only alter the HOMO energy level of **16** in a favourable way, it can also be used for further functionalization. Until now, no silicon rhodamines are known that bear two phenylic halogen substituents (Cl/Cl, Cl/Br, Br/Br nor combinations with F) at the positions adjacent to the xanthene benzene bond. The same holds true for the oxygen counterparts with a dihalogenated pyridinyl motif. In fact, only two pyridinyl silicon rhodamines (**13** and **14**) are known so far, although halogenated pyridines are highly interesting for further functionalization or vector conjugation by nucleophilic aromatic substitution. For the implementation of the dihalogenated pyridine motif into the silicon rhodamine scaffold, we considered using a halogen dance (HD) reaction of 3-bromo-2-chloropyridine (**19**) to **18** followed by a condensation with silicon xanthone **17**, which is accessible in two steps from 3-bromo-*N*,*N*-dimethylaniline. The rearrangement of halo pyridine **19**, initiated by a halogen metal exchange with *n*-BuLi, was initially published and investigated by Mallet et al. who also investigated and termed the mechanism “homotransmetallation” [47]. The HD rearrangement reaction in general is an excellent method for the construction of highly substituted carbo- and heterocyclic systems (e.g., tetrasubstituted pyridines [48]) with substitution patterns difficult to obtain otherwise [49–51].

## Results and Discussion

### Approaches to synthesize the pyridinyl silicon rhodamine **15**

**Scheme 3:**
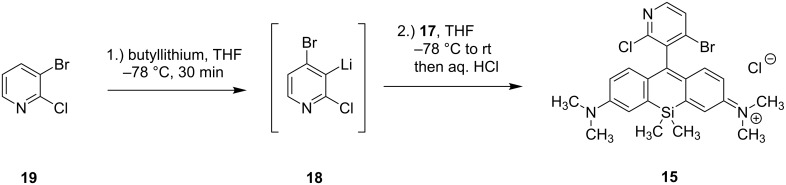
Optimization of the HD rearrangement of **19** and subsequent reaction with xanthone **17** to the silicon rhodamine dye **15**.

Table 2 and Scheme 3 summarize the experimental results towards the synthesis of the radiofluorinatable near-infrared dye **15**. To initiate the HD reaction, 3-bromo-2-chloropyridine (**19**) had first to be lithiated. After 30 min at −78 °C, the silicon xanthone **17** was added at the same temperature and the reaction mixture was subsequently warmed up to room temperature and stirred for varying time periods. By using *t*-BuLi as a lithiation reagent, the desired dye **15** was obtained at just 14% yield as a deep blue solid (Scheme 3, Table 2, entry 1). Owing to the mechanism of the homotransmetallation, the HD rearrangement of **19** is conducted with substoichiometric amounts of the lithiating agent (usually 0.5 equiv *n*-BuLi for 1 equiv **19**) [47,49]. Thus, we tried the reaction with 0.9 equiv of *t*-BuLi (Table 2, entry 2) and 0.5 equiv of *n*-BuLi (Table 2, entry 3), but the desired product was again obtained in poor yields with lots of unreacted starting material **17**. According to the mechanism of the halogen–metal exchange with *t*-BuLi, one equivalent of the base is used for the lithiation, while a second equivalent base eliminates hydrogen bromide from the resulting *t*-BuBr. Therefore, entries 2 and 3 (Table 2) represent the use of approx. 0.5 equiv base for 1 equiv of **19**. After lithiation of **19**, the metallated intermediate (2-chloropyridin-3-yl)lithium reacts again with starting material **19** resulting, after several steps (the so called halogen dance), in the lithiated pyridine intermediate **18** that can add to the silicon xanthone **17**. Low temperatures for the HD reaction are required when more equivalents of the base are used to maintain a coexistence of **19** and its lithiated analogue. Using high excess of **19** could force the reaction to completeness leading to the dihalogenated pyridinyl silicon rhodamine in 85% yield without any monohalogenated byproduct and no necessity of HPLC purification.

**Table 2:** Optimization of the HD rearrangement of **19** and subsequent reaction with xanthone **17** to the silicon rhodamine dye **15**.

Entry	Equiv of lithiation reagent	Equiv of **19**	Yield **15**

1	20 *t*-BuLi	10	14%^a^
2	9 *t*-BuLi	10	14%^b^, 28%^b,c^
3	5 *n*-BuLi	10	11%^d^
4	40 *t*-BuLi	20	85%^e^

^a^1.5 h reaction time after addition of 1 equiv of xanthone **17**; ^b^24 h reaction time; ^c^yield based on recovered starting material of xanthone **17**; ^d^5 h reaction time, ^e^4 h reaction time.

Although **15** can be coupled or further functionalized at the bromine via a nucleophilic substitution, we explored also if the ester analogue *tert*-butyl 5-bromo-6-chloronicotinate could undergo a HD reaction with subsequent xanthone addition. The reaction did not lead to any product, neither with *n*-BuLi nor with *t*-BuLi. However, it is noteworthy that no HD reactions of nicotinic acids can be found in the literature. In fact, if 2,3-dihalogenated pyridines are used for the HD rearrangement as starting materials, only methyl groups are tolerated as carbon substituents.

### Optical properties of the pyridinyl silicon rhodamine **15**

**Figure 1:**
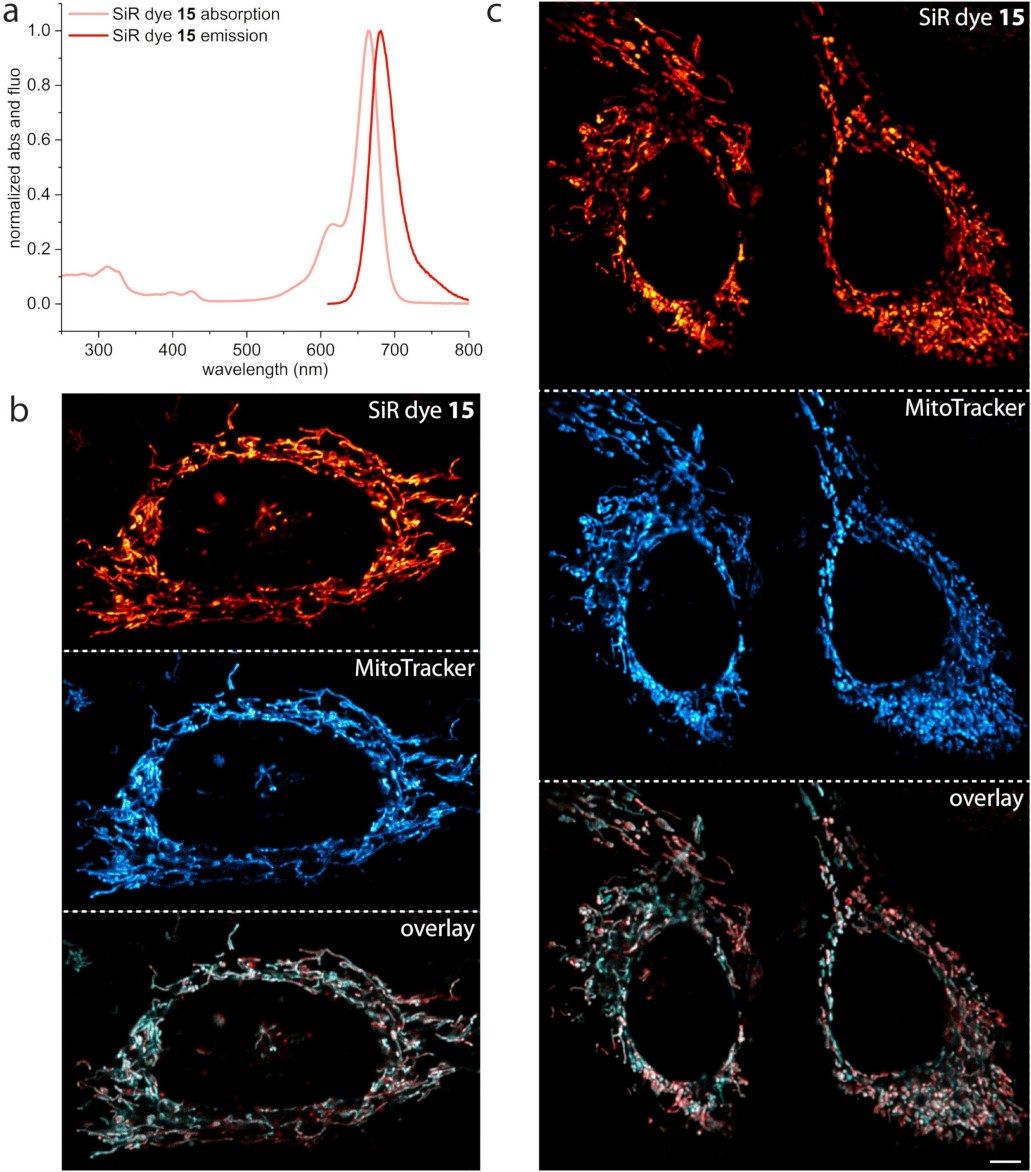
(a) Absorption and fluorescence emission spectra of dye **15** measured in PBS buffer pH 7.4. (b, c) Colocalization experiment of dye **15** (red) and MitoTracker^®^ Green FM (cyan) in living HeLa (b) and U2OS (c) cells supporting the application of dye **15** as a specific NIR mito tracker probe. Both cell lines were incubated for 0.5 h with 1 µM of dye **15** and 100 nM of MitoTracker^®^ Green FM, washed and imaged with excitation at 470 nm (380 µW) and 652 nm (7.5 µW). Confocal images are color shift and background corrected, scale bar 5 µm.

The dihalogenated pyridinyl SiR **15** has an absorption peak at 665 nm (ε_max_ = 34 000 M^−1^cm^−1^) and an emission peak at 681 nm (τ = 1.9 ns, Figure S3a, Table S2a, Supporting Information File 1) (measured in PBS buffer pH 7.4). It shows a red-shift of approx. 10 nm in absorption and emission compared to the azetidine-substituted pyridinyl dye **14** and a 10 nm red-shifted absorption with unchanged emission compared to pyridinyl dye **13** (Figure 1a). The quantum yield is with 0.34 (measured in PBS buffer pH 7.4, Figure S2a, Table S1a, Supporting Information File 1), remarkably higher than the value of pyridinyl dye **13**. This fact could be explained with rotation restriction around the pyridinyl–xanthene bond and/or with beneficial effects of the halogens on the HOMO energy level. Nevertheless, the pyridinyl SiR **14** performs better due to the addition contributions of the azetidine rings.

Next, we examined the targeting ability to mitochondria of the dihalogenated dye **15** by colocalization experiments with the commercially available mitochondria staining reagent MitoTracker^®^ Green FM (Figure 1a,b and Figure S4a,b, Supporting Information File 2). To determine the Pearson coefficient for colocalization of SiR **15** with MitoTracker^®^ Green FM, HeLa cells (human cervical cancer cells) and U2OS cells (human bone osteosarcoma epithelial cells) were co-stained with these dyes. The Pearson coefficients are reasonably high and similar for both cell lines (HeLa cells: 0.85 ± 0.05 (*N* = 20), U2OS cells: 0.81 ± 0.05 (*N* = 27)) supporting the application of SiR **15** as a specific NIR mito tracker probe. Pearson coefficients for selective mitochondria staining >0.8 correlate with much higher lipophilicity (clogP over 4.95). Especially SiR-Mito 8 offers a comparable quantum yield to dye **15** (ε and therefore brightness not available) combined with a Pearson coefficient ≥0.9 [46]. However, dye **15** has the benefit of further red-shifted absorption and emission properties as well as a photostability that allows for STED (stimulated emission depletion) nanoscopy [52–53].

As the 2-chloropyridinyl moiety in SiR dye **15** targets acidic cellular compartments in general, we additionally investigated potential lysosomal colocalization. Co-staining HeLa and U2OS cells with SiR dye **15** and the commercially available lysosomal staining reagent LysoTracker^TM^ Green DND-26 showed absence of any lysosomal targeting ability and confirmed specific mitochondrial staining (Figure S4c,d, Supporting Information File 1).

**Figure 2:**
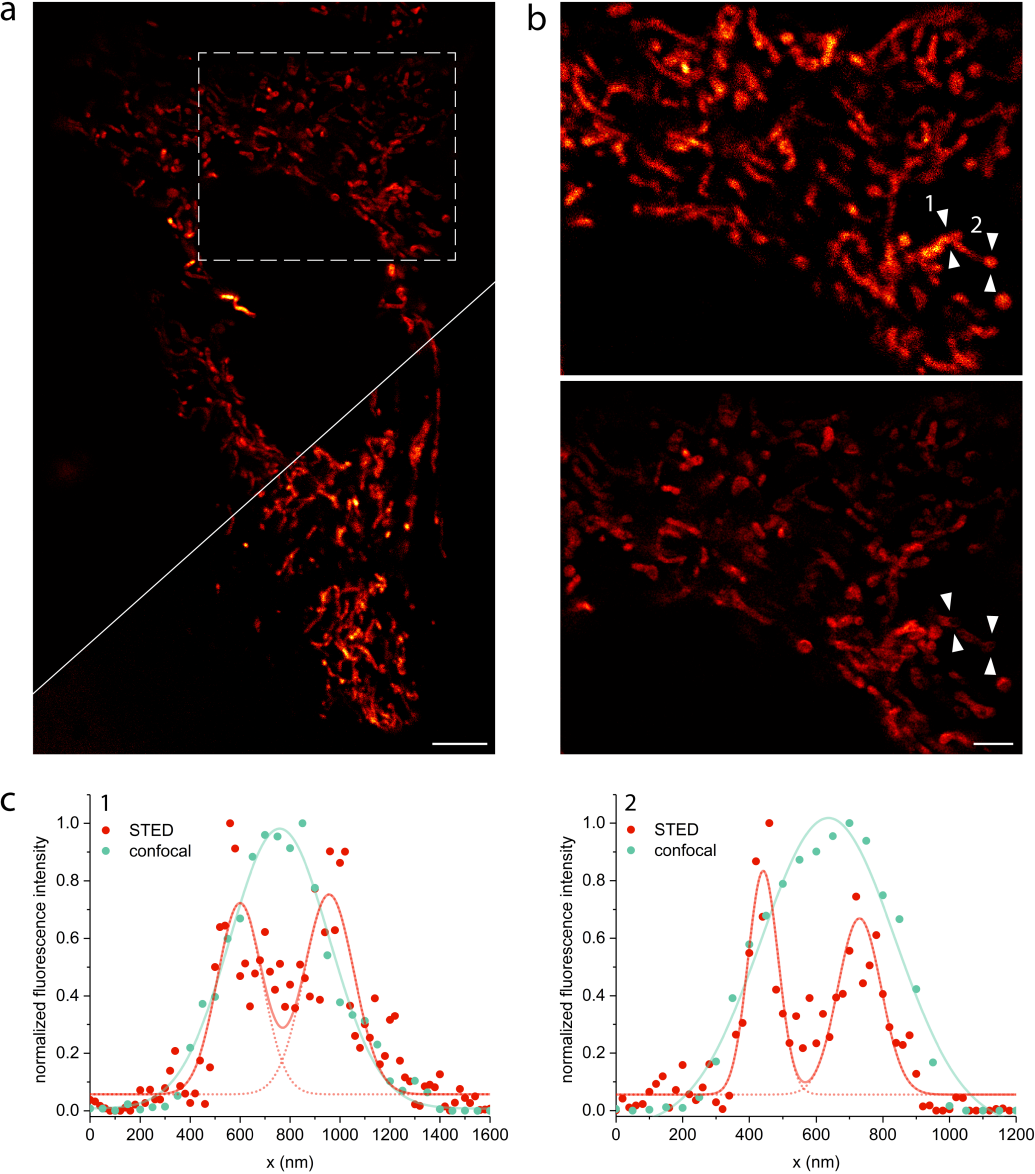
STED and confocal images of the mitochondrial network in living HeLa cells stained with 1 µM SiR dye **15** for 1 h. (a) STED image (excitation at 652 nm with 15 µW, depletion at 775 nm with 40 mW) with the corresponding confocal data (excitation at 652 nm with 7.5 µW) in the bottom right corner. STED image is background subtracted and linearly deconvolved (Lorentzian PSF), confocal image is only background subtracted, scale bar 5 µm. (b) Magnified view of the region marked in (a) in confocal (top) and STED mode (bottom), scale bar 2 µm. (c) Line profiles marked in (b) proving the gain in spatial resolution of STED (red) compared to confocal (cyan). Line profiles were taken from only background corrected (not deconvolved) data (Figure S8, Supporting Information File 1), counts were averaged over five pixels, normalized and fitted to a single (confocal) or double (STED) Gaussian function. To avoid photobleaching, the STED power was set as low as possible but as high as necessary to resolve the tubular structure of mitochondria resulting in full width half maxima (FWHM) of 219 ± 15 nm (1, left), 306 ± 21 nm (1, right), 114 ± 7 nm (2, left) and 200 ± 9 (2, right). The peak to peak distances are 363 nm (1) and 294 nm (2). The FWHM of the confocal fits are 470 ± 15 nm (1) and 474 ± 25 nm (2).

Medical imaging agents are highly interesting especially if they can address multiple questions or can be applied for different purposes simultaneously, because they must undergo an expensive regulatory process before they attain approval for the market. Therefore, we are interested also in purposes other than PET imaging or macroscopic fluorescence imaging. Histopathological examinations of biopsy material on subcellular level need high image quality. Thus, the option to use our proposed bimodal dye in STED nanoscopy would be advantageous. For example, Giedt et al. could show that analysis of mitochondrial morphology can be used as a biomarker for cancer phenotype assignment and for drug response analysis [54]. For STED imaging, HeLa cells were stained for 1 h with 1 µM of dye **15**, washed and imaged live. Figure 2 and Figure S5, Supporting Information File 1, compare STED images with their corresponding confocal images. By resolving the tubular structure of mitochondria, we prove successful application of our mitochondria-selective pyridinyl SiR **15** in STED nanoscopy.

### Toxicity of the pyridinyl silicon rhodamine **15**

**Figure 3:**
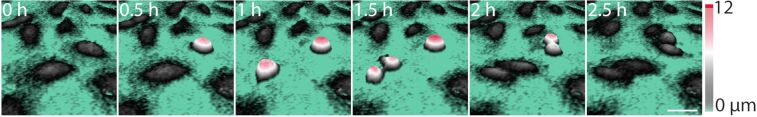
Exemplary holographic image sequence of two cell divisions of U2OS cells treated with 1 µM of dye **15**. Dividing cells round up and can be distinguished from non-dividing cells by height. After incubation with 1 µM dye **15** for 1 h, the cells were washed and then holographically imaged using a HoloMonitor^®^ M4 time-lapse cytometer. Cell proliferation was followed for 14.5 h (30 min between images) and corresponding time-lapse movies are available in Supporting Information Files 2–5, scale bar 50 µm.

Although for PET examinations only nano- or picomolar amounts of the radiopharmaceutical compound are needed, medical applications of fluorescence dyes (e.g., fluorescence-guided interventions) require larger amounts of material. Therefore, cytotoxicity testing is necessary for our proposed bimodal imaging agent **16** and its precursor **15**. For toxicity assessment, the frequency and duration of cell division with and without incubation with dye **15** was analyzed via time-lapse holographic imaging (Figure 3). U2OS cells were incubated with 1 µM dye **15** in medium for 1 h and, after washing with dye free medium, continually imaged over a period of 14.5 h using a holographic incubator microscope. The analysis of the data revealed that the frequency and duration of cell division of the cells incubated with dye **15** show no difference to the untreated control (frequency of cell division with SiR dye **15**: 0.30 ± 0.05 divisions per cell, without dye: 0.32 ± 0.06 divisions per cell, for division duration see Figure S7, Supporting Information File 1). These results are supported by cell count and confluency analysis (Figure S9, Supporting Information File 1). In summary, we conclude that dye **15** does not show any significant cytotoxicity in this human cell line. Comparable experiments with HeLa cells strengthen these results (data not shown).

## Conclusion

We have proven the feasibility of synthesizing a pyridinyl silicon rhodamine dye with two halogen atoms adjacent to the xanthene–pyridine bond by application of a halogen dance rearrangement. By our optimized procedure, we have obtained the dye **15** at high yield and without the necessity of HPLC purification. The chlorine atom in 2’-position can potentially be used to introduce the PET radionuclide fluorine-18 while the bromine atom serves as a constraint against rotation around the xanthene–pyridine bond as well as a leverage point for further linkage. The quantum yield is reasonably high (0.34). However, despite the improved molecule rigidity and thus presumably less nonradiative decay, dye **15** does not outperform the quantum yield of monosubstituted pyridine SiR **14**. Additional experiments (supported by DFT calculations) on the orbital effects of both halogens and the nitrogen position in the pyridine ring are needed to explain these effects with confidence.

In addition, our SiR dye **15** displays photophysical properties (extinction coefficient, quantum yield, lifetime) in the same range, but rather at the lower end, compared to other near-infrared silicon rhodamine derivatives with similar spectral properties [3–4,7]. However, it is in line with the group of SiR derivatives directly targeting certain cellular structures or organelles [7,55–57] and extends this list with a NIR mito tracker**^®^** probe.

Just like the recently published squaraine variant dye MitoESq-635 [58], our SiR dye **15** offers the option of imaging mitochondria in living cells using STED nanoscopy without the necessity of an additional tagging step. In contrast to MitoESq-635, our SiR dye **15** selectively stains mitochondria without background from unspecific membrane staining. However, higher photostability and a lower saturation intensity for STED result in a better performance in time-lapse live cell STED imaging of MitoESq-635. Taken together, our SiR dye **15** is a valid compromise between MitoESq-635 and SiR-Mito 8 offering nontoxic, specific mitochondrial staining in live cell STED imaging.

In summary, we present a biocompatible, nontoxic, small molecule near-infrared dye with the option of subsequent radiolabelling and excellent optical properties for biomedical imaging. As a compound with intrinsic mitochondria targeting ability, the radiolabelled analogue can find application in multimodal (PET/OI) imaging of mitochondria for diagnostic and therapeutic use in, e.g., cancer patients. (Radio)fluorination of dye **15** is the subject of ongoing research and will be presented elsewhere.

## Supporting Information

Synthesis of dye **15**, its optical characterization and detailed information on microscopy experiments, including videos showing undisturbed cell proliferation in U2OS cells incubated with 1 µM of dye **15** for 1 h compared to untreated U2OS cells are given.

File 1 Experimental and analytical data, spectra, live cell imaging and assessment of cytotoxicity.

File 2 Independent experiment assessing cell division of U2OS cells after treatment with 1 µM SiR dye **15**.

File 3 Independent experiment assessing cell division of U2OS cells after treatment with 1 µM SiR dye **15**.

File 4 Independent control experiment assessing undisturbed cell division of U2OS cells.

File 5 Independent control experiment assessing undisturbed cell division of U2OS cells.
