# Supplementary material for: Synthesis of a dihalogenated pyridinyl silicon rhodamine for mitochondrial imaging by a halogen dance rearrangement
Source: Beilstein J Org Chem. 2019 Oct 1;15:2333–43. doi: 10.3762/bjoc.15.226 (PMC6808212; doi:10.3762/bjoc.15.226)
Supplement: File 1 — Experimental and analytical data, spectra, live cell imaging and assessment of cytotoxicity. [file Beilstein_J_Org_Chem-15-2333-s001.pdf]

BEILSTEIN JOURNAL OF ORGANIC CHEMISTRY

Supporting Information

for

Synthesis of a dihalogenated pyridinyl silicon rhodamine for mitochondrial imaging by a halogen dance rearrangement

Jessica Matthias, Thines Kanagasundaram, Klaus Kopka and Carsten S. Kramer

Beilstein J. Org. Chem. **2019,** *15,* 2333–2343. doi:10.3762/bjoc.15.226

Experimental and analytical data, spectra, live cell imaging and assessment of cytotoxicity

License and Terms: This is a supporting information file under the terms of the Creative Commons Attribution License (http://creativecommons.org/licenses/by/4.0). Please note that the reuse, redistribution and reproduction in particular requires that the authors and source are credited.
The license is subject to the *Beilstein Journal of Organic Chemistry* terms and conditions: (https://www.beilstein-journals.org/bjoc)

Table of contents

1. General remarks.......... S2

2. Synthesis of SiR dye **15** S3

3. NMR data of dye 15.......... S4

^{1}H NMR spectrum (400 MHz, methanol-d_4) of **15**.......... S4

^{13}C NMR spectrum (101 MHz, methanol-d_4) of **15**.......... S4

4. Fluorescence properties S5

4.1 Absorption and emission.......... S5

4.2 Fluorescence quantum yield S5

4.3 Fluorescence lifetime S7

5. Live cell imaging.......... S9

5.1 Colocalization experiments S10

5.2 STED imaging.......... S14

6. Assessment of cytotoxicity.......... S16

1. General remarks

Not otherwise described, reactions requiring exclusion of oxygen and moisture were carried out in heat-gun flasks dried under argon gas atmosphere using the Schlenk technique.

All **chemicals** and **solvents** were used from Sigma-Aldrich Laborchemikalien GmbH, abcr GmbH, Acros Organics and were used as received without other purification. Deuterated solvents were used from Deutero GmbH.

NMR spectra were recorded at room temperature on a 400 MHz Bruker Avance III spectrometer. Chemical shifts are reported in *δ* units relative to methanol-d_4 (δ_H = 3.31; δ_C = 49.0).[1] Analyses followed first order and the following abbreviations were used throughout: s = singlet, d = doublet, t = triplet, dd = doublet of doublet etc., m = multiplet. Coupling constants (*J*) are given in Hz and refer to H,H-couplings. Assignment (C_{quart}, CH, CH_2, CH_3) was conducted with DEPT and HSQC experiments.

Mass spectra (MS) were determined in the organic chemistry department of the University of Heidelberg under the direction of Dr. Jürgen Gross. The ionization method ESI was applied using spectrometer BrukerApexQe hybrid 9.4 T FT-ICR. High-resolution mass spectra (HRMS) were recorded on a JEOL JMS-700 spectrometer. The molecule fragments are reported in mass to charge (*m/z*) relation.

Spectroscopic characterization and cell experiments of/with SiR dye **15** were done in the Max Planck Institute for Medical Research Heidelberg, Department of Optical Nanoscopy by M. Sc. Jessica Matthias.

Analytical thin layer chromatography (TLC) was carried out on polygram-DC-plates produced by Machery-Nagel (40 × 80 mm, SIL G/UV_{254}, 0.2 mm layer thickness). Detection was carried out using UV-light (254 nm or 366 nm). TLC on reversed-phase were carried out on alugram-DC-plates produced by Machery-Nagel (40 × 80 mm, RP-18 W/UV_{254}, 0.15 mm layer thickness).

Flash column chromatography was carried out on silica gel (0.032 mm–0.062 mm, produced by Macherey-Nagel) using manual techniques.

For drawing **chemical formulas** and performing **cLogP calculations** ChemDraw Professional 16.0.1.4 was used.

[1] G. R. Fulmer, A. J. M. Miller, N. H. Sherden, H. E. Gottlieb, A. Nudelman, B. M. Stoltz, J. E. Bercaw, K. I. Goldberg, *Organometallics* **2010**, *29*, 2176–2179.

2. Synthesis of SiR dye 15

In a flame-dried flask, 3-bromo-2-chloropyridine **19** (249 mg, 1.29 mmol, 20 equiv) was dissolved in 3.0 mL anhydrous THF under argon. The solution was cooled to −78 °C, then *tert*-BuLi (1.7 M in pentane, 2.59 mmol, 1.52 ml, 40 equiv, CAUTION: solutions of *tert*-butyllithium react explosively with water and may ignite in moist air) was added drop-wise and the brown-red mixture was stirred for 30 min. At the same temperature, silicon xanthone **17**[2] dissolved in anhydrous THF (2.0 mL) was added drop-wise. The mixture went deep red. The mixture was warmed to room temperature after 2 min and the mixture turned dark. The reaction was stirred for 4 hours, then water and DCM were added. Some drops of 1 N HCl were added and the organic phase turned deep blue. The organic phase was separated and the water phase was extracted with DCM several times. The organic phase was dried with brine and sodium sulfate and after filtration, the solvents were evaporated in vacuo. Column chromatography (silica gel, 1% to 10% methanol in DCM) afforded **15** as a deep blue solid (29.5 mg, 0.0551 mmol) in 85% yield.

^{1}H NMR (400 MHz, methanol-d_4) δ = 8.55 (d, *J*=4.8, 1H), 7.41 (d, *J*=2.8, 2H), 7.38 (d, *J*=4.8, 1H), 7.01 (d, *J*=9.7, 2H), 6.84 (dd, *J*=9.6, 2.9, 2H), 3.38 (s, 12H), 0.63 (s, 3H), 0.60 (s, 3H).

^{13}C NMR (101 MHz, methanol-d_4) δ = 163.4 (C_{quart}), 155.9 (C_{quart}), 153.55 (C_{quart}), 153.3 (C_{quart}), 149.4 (C_{quart}), 149.0 (CH), 141.2 (CH), 126.69 (C_{quart}), 126.0 (CH), 123.0 (CH), 122.1 (C_{quart}), 115.8 (CH), 41.1 (CH_3), -1.0 (CH_3), -1.5 (CH_3).

HRMS (ESI) calcd. for $C_{24}H_{26}BrClN_3Si$ [M^+] 498.0768, found 498.0772.

R_f = 0.44 (methanol : DCM = 1 : 9)

---

[2] G. Lukinavicius, K. Umezawa, N. Olivier, A. Honigmann, G. Yang, T. Plass, V. Mueller, L. Reymond, I. R. Correa Jr, Z.-G. Luo, C. Schultz, E. A. Lemke, P. Heppenstall, C. Eggeling, S. Manley, K. Johnsson, *Nat. Chem.* **2013**, *5*, 132–139.

3. NMR data of dye 15

^{1}H NMR spectrum (400 MHz, methanol-*d*4) of 15

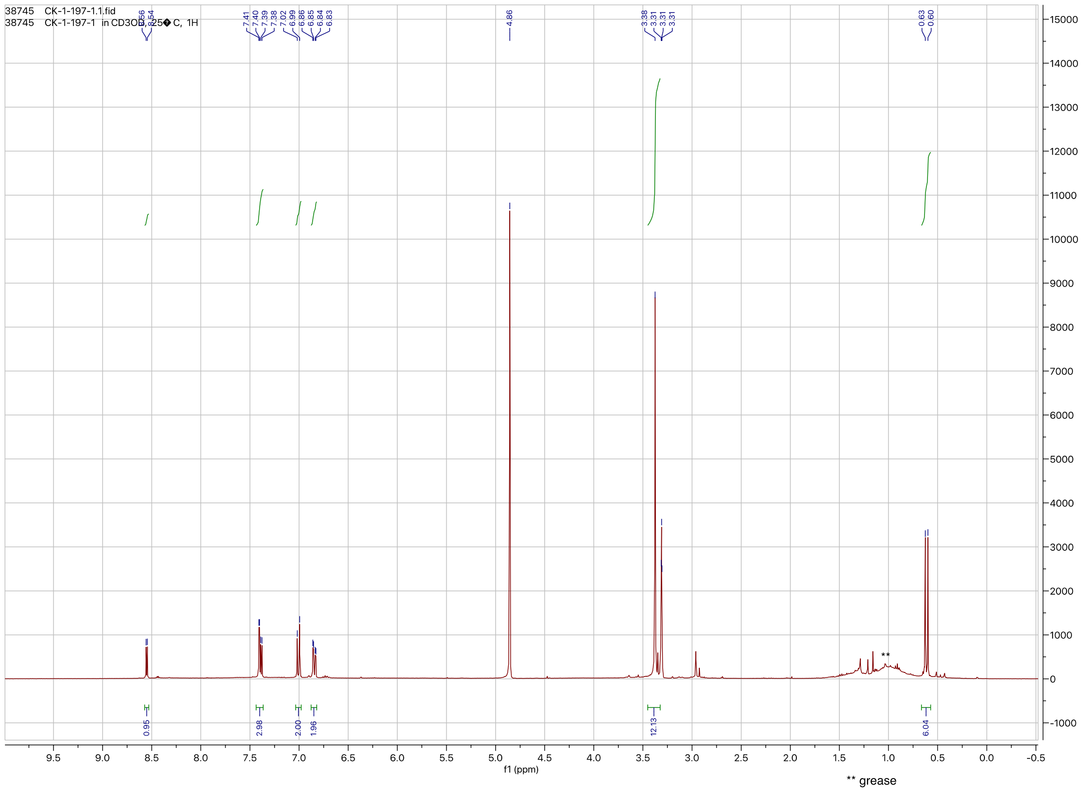

^{13}C NMR spectrum (101 MHz, methanol-*d*4) of 15

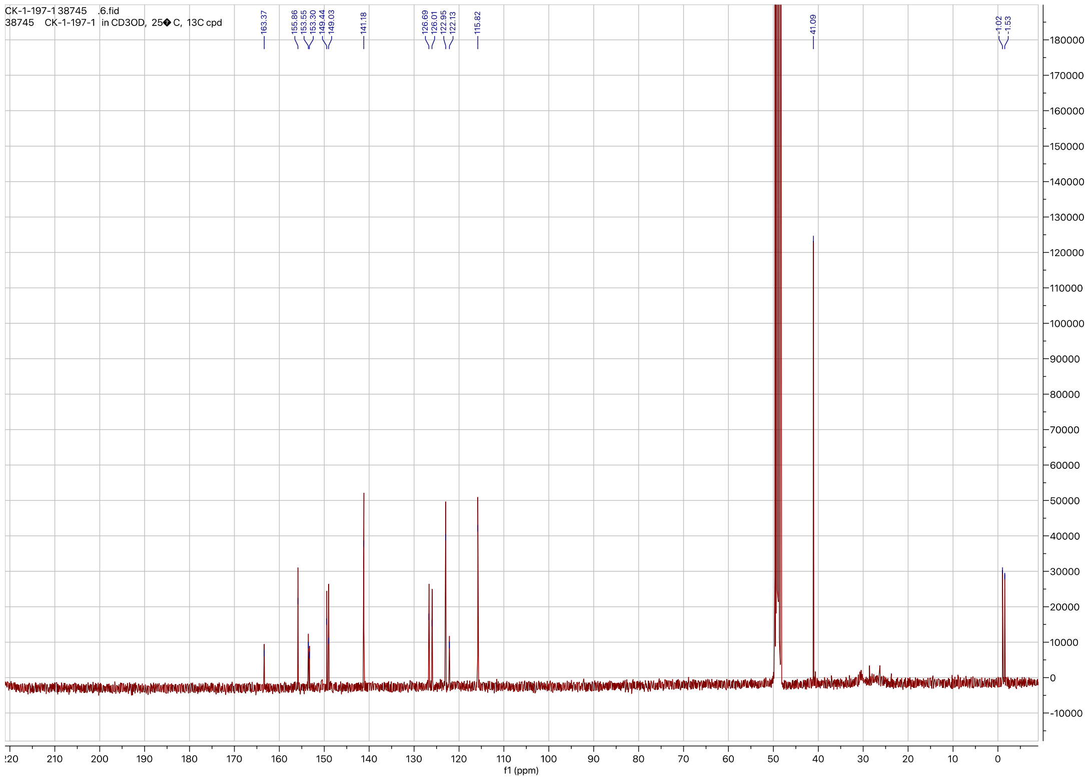

4. Fluorescence properties

4.1 Absorption and emission

Absorbance spectra were recorded on a Cary 5000 UV–vis-NIR spectrophotometer, emission spectra on a Cary Eclipse Fluorescence spectrophotometer with excitation at 600 nm using standard 1 cm quartz cuvettes. The stock solution of dye **15** (4.67 mM in MeCN) was diluted in either PBS pH 7.4 or in MeCN to 15.5 µM for UV–vis/NIR absorbance measurements or to 4.7 µM for fluorescence emission measurements. The extinction coefficient ε_{max} was calculated according to Lambert–Beer for the absorbance maximum.

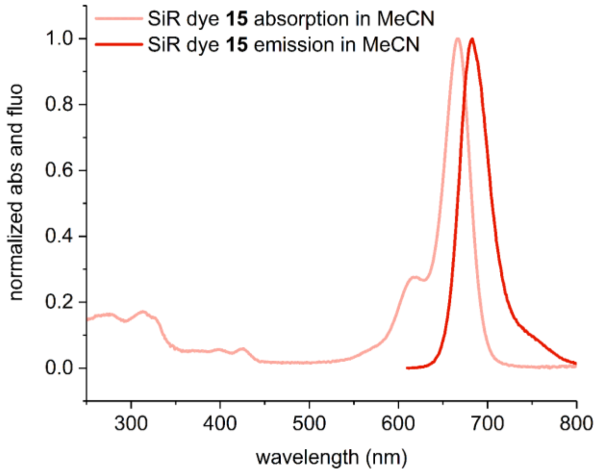

Figure S1: Absorption (abs) and emission (fluo) spectrum of dye **15** in MeCN with absorption and emission maximum at 667 nm and 683 nm, respectively.

4.2 Fluorescence quantum yield

Fluorescence quantum yield was determined via relative measurements according to the literature procedure[3,4] using the reference standard dye Nile blue (ϕ_{ref} = 0.27)[5]. The stock solutions of SiR dye **15** (4.67 mM in MeCN) and Nile Blue (1 mM in DMSO) were diluted to the following dilution series considering an absorbance between 0.01 and 0.1 at the excitation wavelength of 600 nm:

SiR dye **15**: 4 to 5 dilutions between 0.8 µM – 4.7 µM in PBS pH 7.4 or MeCN

Nile blue: 4 to 5 dilutions between 0.2 µM – 1.0 µM in 5% (v/v) 0.1 M HCl in EtOH

[3]C. Wurth et al., *Nat. Protoc.*, **2013**, 8, 8, 1535–1550.
[4]A. T. R. Williams, S. A. Winfield, J. N. Miller, *Analyst* **1983**, *108*, 1290, 1067–1071.
[5]R. Sens, K. H. Drexhage, *Journal of Luminescence* **1981**, *24-5,* 709–712.

Absorbance of each dilution was recorded on a Cary 5000 UV–vis-NIR spectrophotometer in the 200–800 nm interval. Afterwards, emission of the same sample was recorded on a Cary Eclipse Fluorescence spectrophotometer with excitation at 600 nm in the 610–800 nm interval. All dilutions were measured using the same parameters. Absorbance at 600 nm was plotted versus the integrated fluorescence intensity and fitted by linear regression (Figure S2). The fit values (Table S1) of the slopes were used to calculate the fluorescence quantum yield according to

$$\phi = \phi_{ref}\left(\frac{m}{m_{ref}}\right)\left(\frac{n}{n_{ref}}\right)^2$$

with ϕ (ϕ_{ref}) being the fluorescence quantum yield, m (m_{ref}) the slope of the linear fit and n (n_{ref}) the refractive index of the solvent of the sample (reference standard).

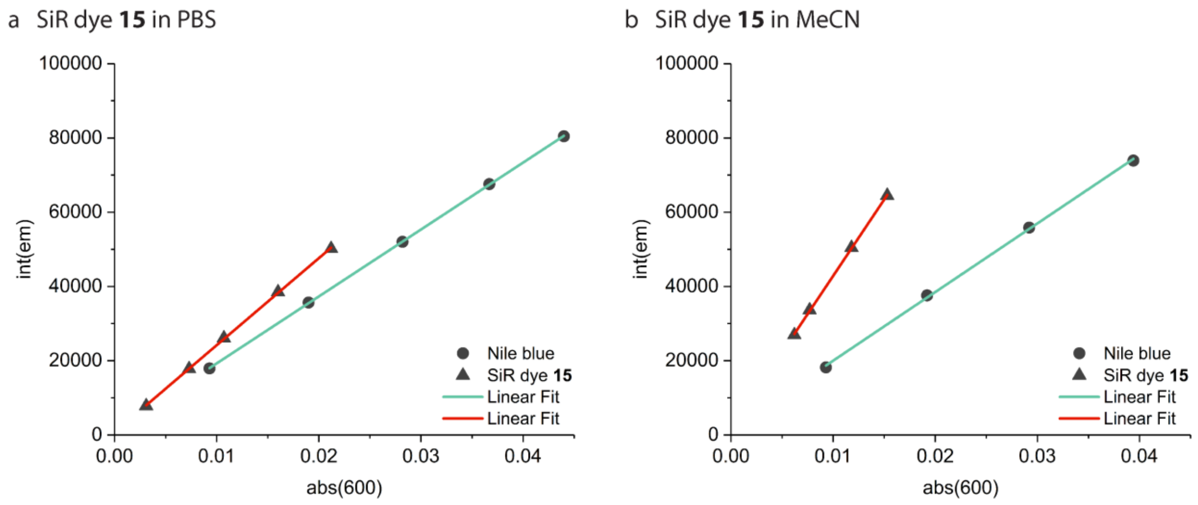

Figure S2: Relative measurement of the quantum yield of dye **15** in PBS pH 7.4 (a) and in MeCN (b) using Nile Blue as reference standard.

Table S1: Fit parameter for the relative measurement of the quantum yield of dye **15** in PBS pH 7.4 (a) and in MeCN (b) using Nile blue as reference standard.

a

	SiR dye **15** in PBS pH7.4	Nile blue
Equation	$y = a + mx$	
Weight	No Weighting	
Intercept a	743.15799 ± 273.17765	1248.8867 ± 179.01284
Slope m	2.34389E6 ± 20558.50971	1.8028E6 ± 5950.1804

Residual Sum of Squares	257461.19762	80813.089
Pearson's r	0.99988	0.99998
R-Square (COD)	0.99977	0.99997
Adj. R-Square	0.99969	0.99996

b

	SiR dye **15** in MeCN	Nile Blue
Equation	$y = a + mx$	
Weight	No Weighting	
Intercept a	1699.91734 ± 470.15064	1517.18908 ± 818.07845
Slope m	4.11367E6 ± 43324.0369	1.84844E6 ± 30593.70394
Residual Sum of Squares	190737.91222	941642.04753
Pearson's r	0.99989	0.99973
R-Square (COD)	0.99978	0.99945
Adj. R-Square	0.99967	0.99918

4.3 Fluorescence lifetime

Fluorescence lifetime was measured with a FluoTime 300 Fluorescence Lifetime Spectrometer by time correlated single photon counting (TCSPC). The stock solution of dye **15** (4.67 mM in MeCN) was diluted in either PBS pH 7.4 or in MeCN to 4.7 µM. The sample was excited at 640 nm and the fluorescence intensity decay $I(t)$ was fitted using the monoexponential fit function

$$I(t) = \int_{-\infty}^{t} IRF(t') \sum_{i=1}^{n} A_i e^{-\frac{t-t'}{\tau_i}} dt'$$

with τ being the fluorescnece lifetime, IRF the instrument response function and A the amplitude of the exponential decay (Figure S3, Table S2).

a

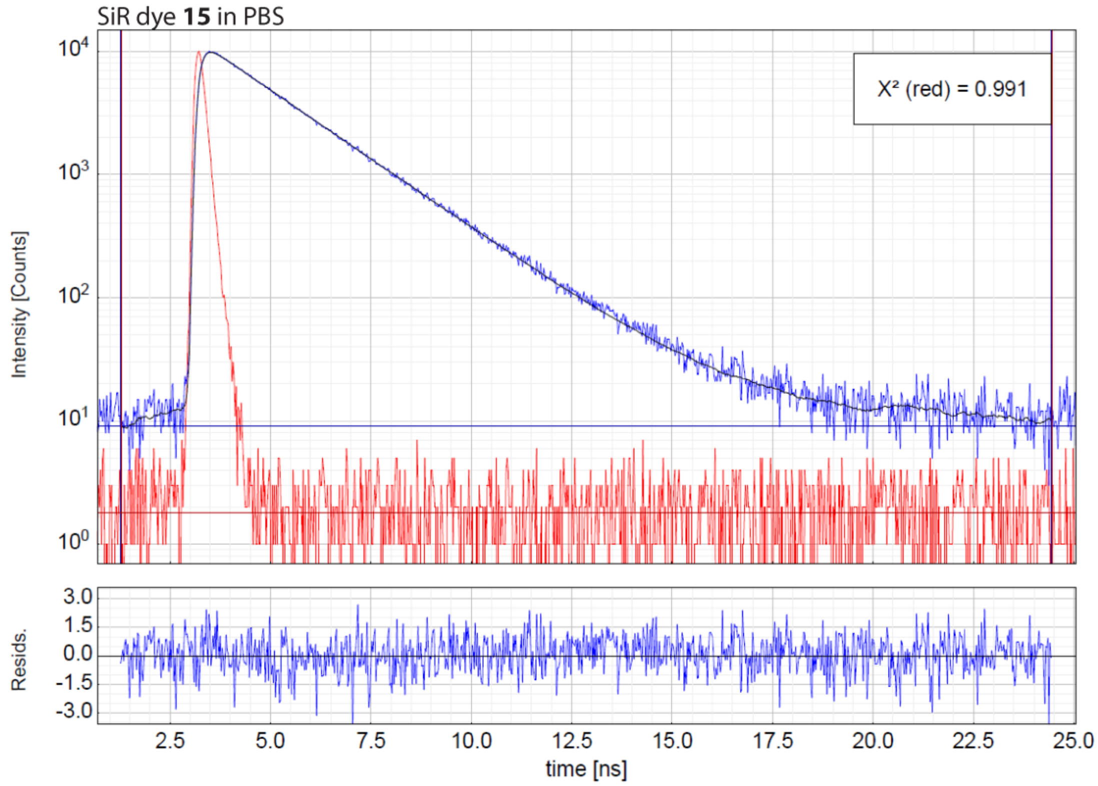

b

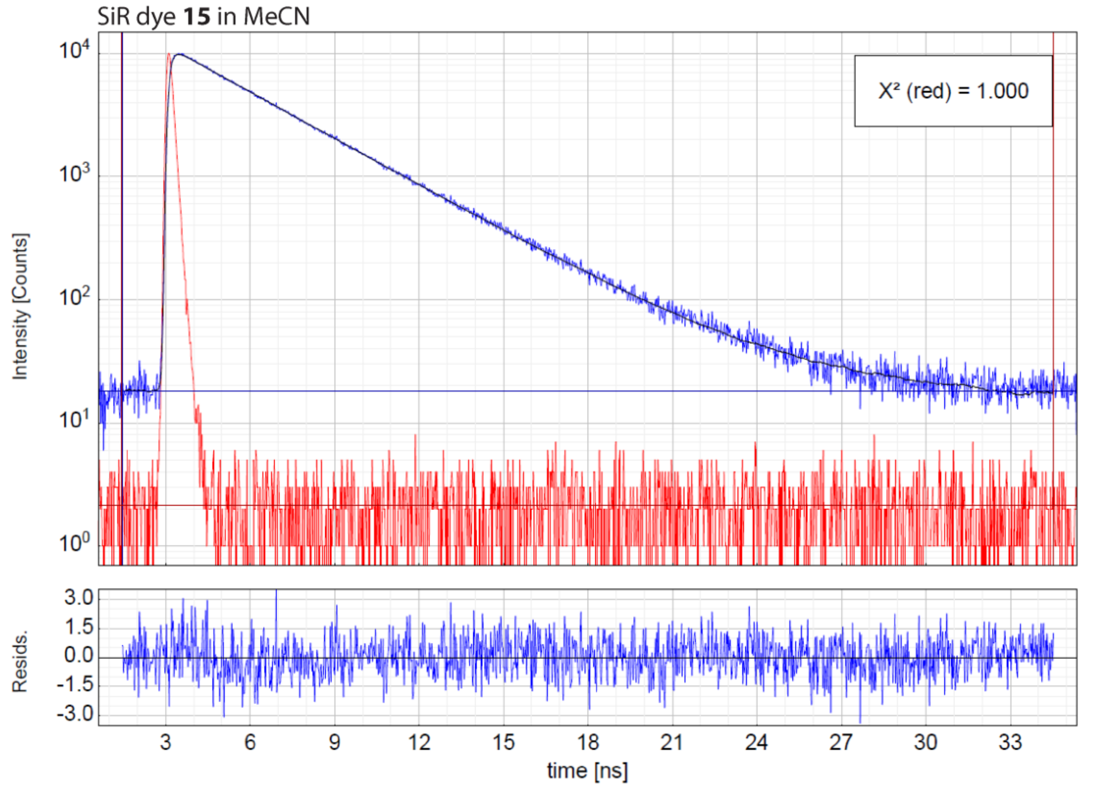

Figure S3: Fluorescence decay of SiR dye **15** (blue) in PBS pH 7.4 (a) and in MeCN (b) fitted with a monoexponential fit function to determine the fluorescence lifetime. Instrument response function (IRF) is shown in red.

Table S2: Fit Parameter for the monoexponential fit of the fluorescence decay of SiR dye **15** in PBS pH 7.4 (a) and in MeCN (b).

a

	SiR dye **15** in PBS pH7.4
A [counts]	12251.4 ± 61.3
τ [ns]	1.93329 ± 0.00704
Background decay [counts]	9.143 ± 0.861
Background IRF [counts]	1.793 ± 0.111
Shift IRF [ns]	0.02126 ± 0.00216
A Scat [counts]	3390 ± 1260
χ^2	0.991

b

	SiR dye **15** in MeCN
A [counts]	11556.5 ± 54.1
τ [ns]	3.4247 ± 0.0118
Background decay [counts]	18.04 ± 1.19
Background IRF [counts]	2.1459 ± 0.0925
Shift IRF [ns]	-0.01255 ± 0.00288
A Scat [counts]	6040 ± 1550
χ^2	1.000

5. Live cell imaging

Samples were imaged on a custom-built STED microscope similar to the one published by Görlitz et al., 2014[6] with a supercontinuum laser (Solea from PicoQuant) and a pulsed 470 nm diode laser (LDH-D-C470 from PicoQuant) for excitation combined with a 775 nm STED laser (nanosecond pulsed fiber laser from MPBC).

[6]F. Görlitz, et al., *Progress in Electromagnetics Research-Pier* **2014,** *147,* 57–68.

5.1 Colocalization experiments

U2OS (human bone osteosarcoma epithelial) and HeLa (human cervical cancer) cells were seeded one day prior to the imaging experiment in 8-well Lab-Tek II chambered coverglass. For assessing mitochondrial colocalization, cells were stained for 30 min with 1 µM SiR dye **15** and 100 nM MitoTracker® Green FM in FluoroBrite™ DMEM at 37 °C and 5% CO_2, washed three times with prewarmed FluoroBrite™ DMEM and imaged immediately live. Confocal images were recorded exciting the SiR dye **15** at 652 nm with 7.5 µW and the MitoTracker® Green FM at 470 nm with 380 µW using a pixel size of 50 nm, a dwell time of 10 µs and a pinhole of 125 µm. For assessing lysosomal colocalization, cells were stained for 30 min with 1 µM SiR dye **15** at 37°C and 5% CO_2, washed three times with prewarmed FluoroBrite™ DMEM and imaged immediately live in FluoroBrite™ DMEM supplemented with 500 nM LysoTracker™ Green DND-26. Confocal images were recorded exciting the SiR dye **15** at 652 nm with 7.5 µW and the LysoTracker™ Green DND-26 at 470 nm with 60 µW using a pixel size of 50 nm, a dwell time of 20 µs and a pinhole of 125 µm. First, confocal images were corrected for color shift between the two channels using a home-written ImageJ[7] routine. In this routine, the images are correlated using the plugin *FD Math*, and shifted based on the found maxima with the plugin *Find Maxima*. Next, the Pearson correlation coefficient was calculated using the ImageJ plugin *JACoP*[7,8]. All confocal dual-color images shown in the main text (Figure 1) and in the SI (Figure S4) were background corrected (subtraction of max. 10% of the maximum signal) additionally.

[7]C. A. Schneider, W. S. Rasband, K. W. Eliceiri, *Nat. Methods* **2012**, *9*, 7, 671–675.
[8]S. Bolte, F. P. Cordelieres, *Journal of Microscopy-Oxford* **2006**, *224*, 213–232.

a

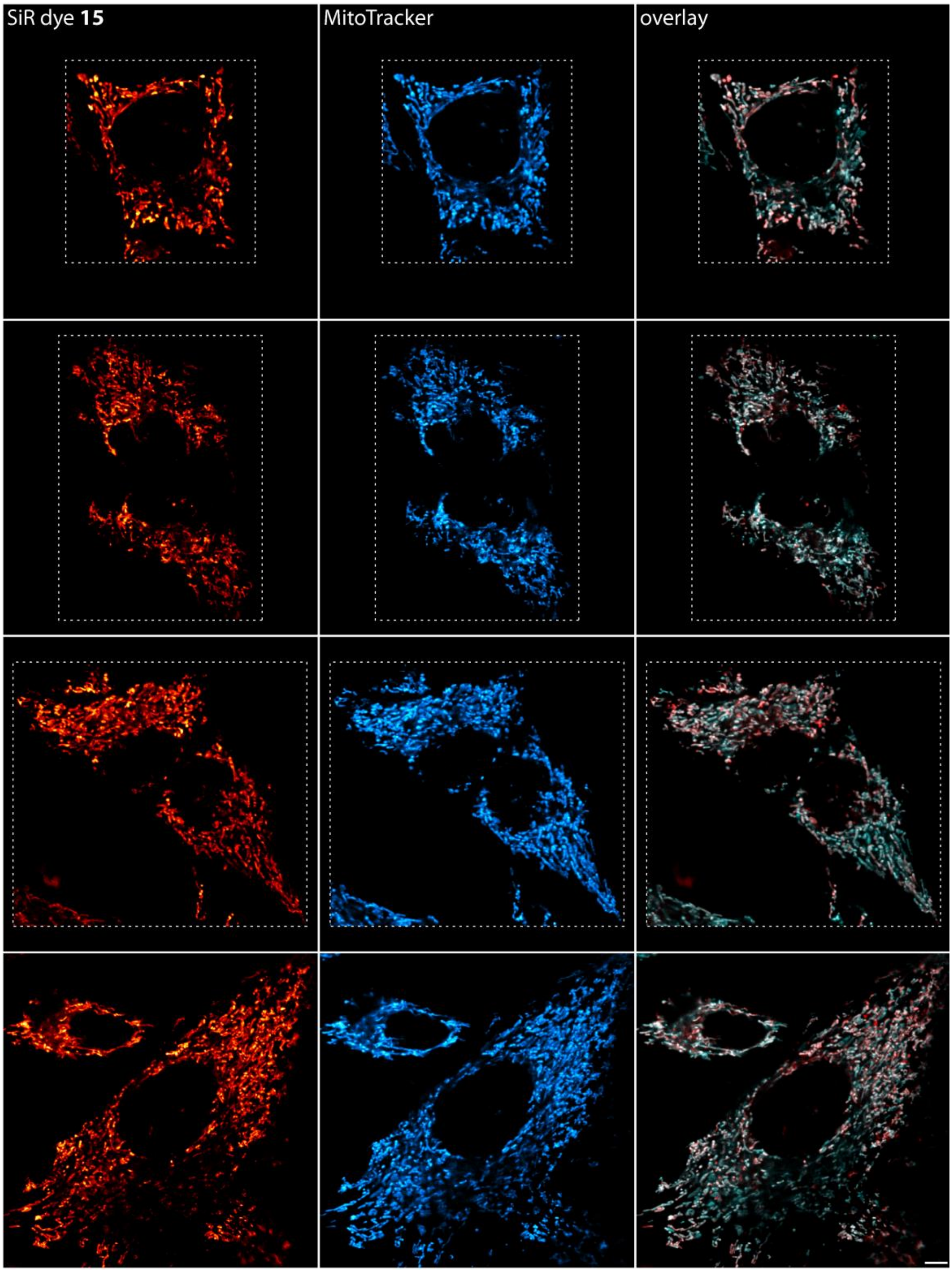

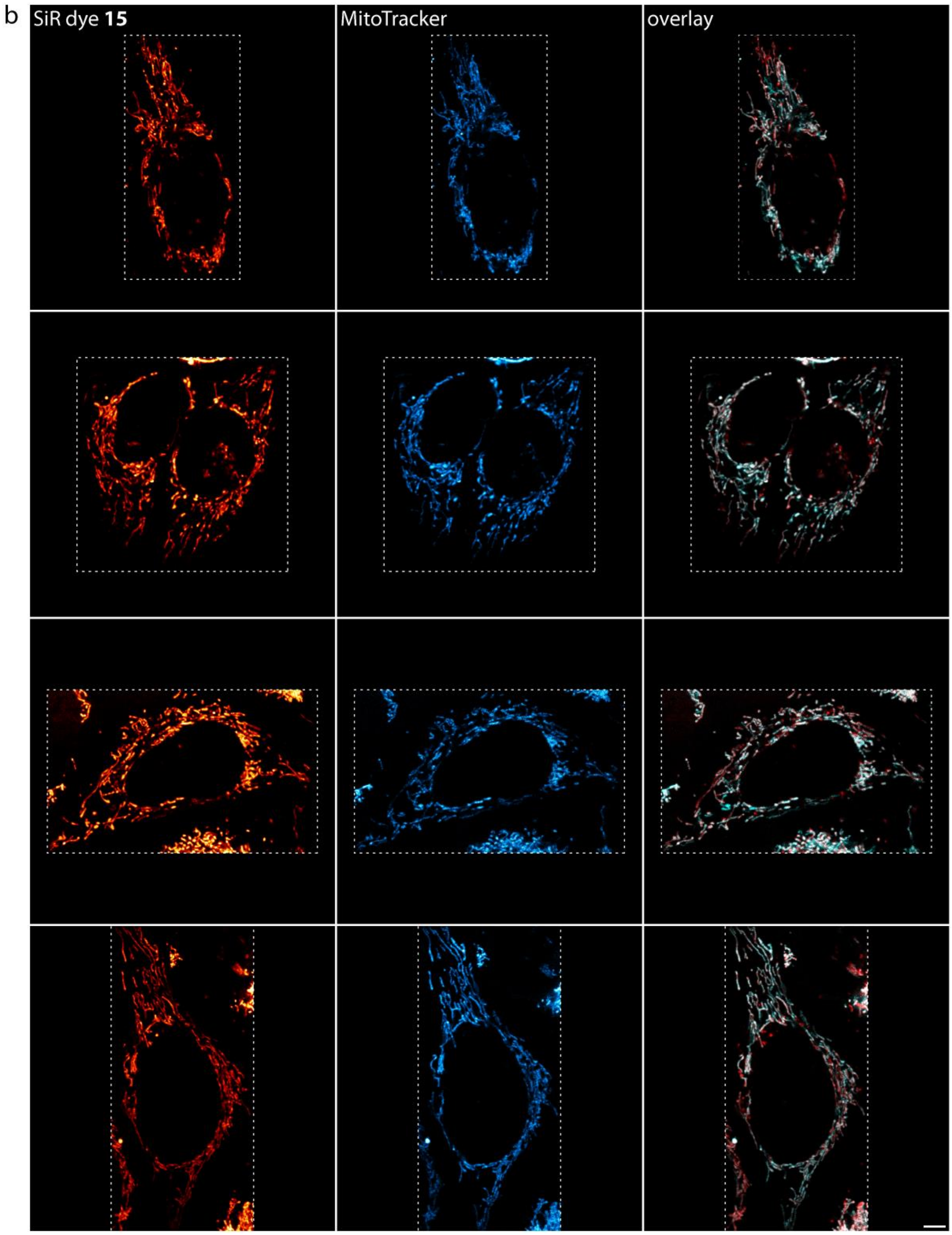
b
SiR dye 15
MitoTracker
overlay

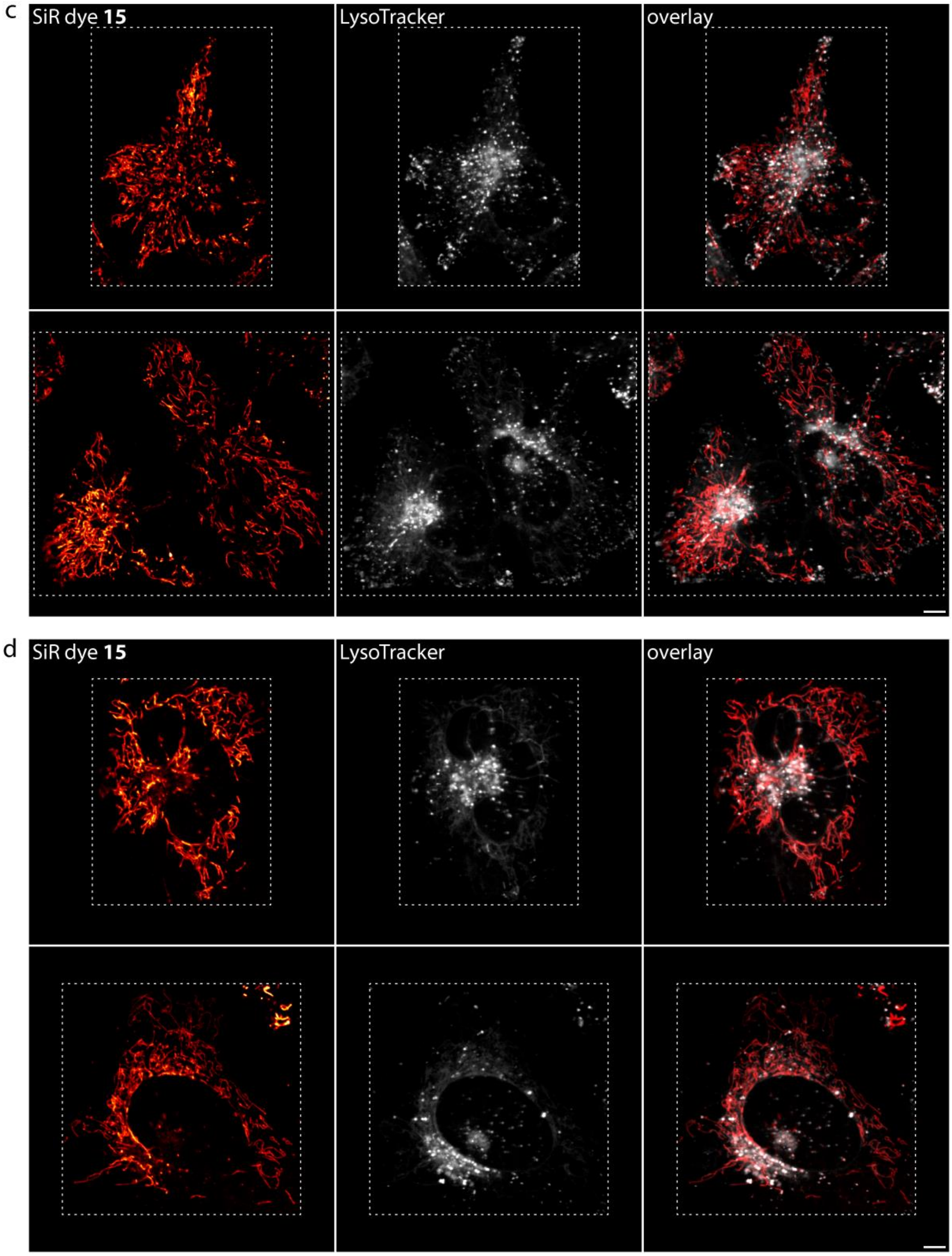

Figure S4: Colocalization experiments of SiR dye **15** (red) with MitoTracker® Green FM (cyan) or LysoTracker™ Green DND-26 (gray). (a, b) Confocal imaging confirms strong mitochondrial colocalization of SiR dye **15** in living HeLa (a) and U2OS (b) cells (Pearson correlation coefficient HeLa cells: 0.85 ± 0.05 (N = 20), U2OS cells: 0.81 ± 0.05 (N = 27)). Both cell lines were incubated for 0.5 h with 1 µM of dye **15** and 100 nM of MitoTracker® Green FM, washed and imaged with excitation at 470 nm (380 µW) and 652 nm (7.5 µW). (c, d) Confocal imaging proves the absence of lysosomal targeting ability of SiR dye **15** in living HeLa (c) and U2OS (d) cells thereby confirming specific

mitochondrial staining. The Pearson correlation coefficients for lysosomal colocalization (HeLa cells: 0.35 ± 0.06 (N = 12), U2OS cells: 0.38 ± 0.04 (N = 13)) overestimate lysosomal targeting affinity of SiR dye **15** as LysoTracker™ Green DND-26 shows mild but additional mitochondria staining. Both cell lines were incubated for 0.5 h with 1 µM of dye **15**, washed and imaged in the presence of 500 nM LysoTracker™ Green DND-26 with excitation at 470 nm (60 µW) and 652 nm (7.5 µW). Confocal images are color shift corrected and background subtracted, scale bar 5 µm.

5.2 STED imaging

HeLa (human cervical cancer) cells were seeded one day prior to the imaging experiment in 8-well Lab-Tek II chambered coverglass. Cells were stained for 1 h with 1 µM SiR dye **15** in FluoroBrite™ DMEM at 37 °C and 5% CO_2, washed three times with prewarmed FluoroBrite™ DMEM and imaged immediately live. STED (confocal) images were recorded exciting the SiR dye **15** at 652 nm with 15 µW (7.5 µW) and depleting at 775 nm with 40 mW (0 mW) using a pixel size of 20 nm, a dwell time of 10 µs and a pinhole of 125 µm. The STED images were, unless otherwise stated, background corrected (subtraction of max. 10% of the maximum signal) and linearly deconvoled using a Lorentzian point spread function (PSF) with a full width half maximum (FWHM) of 40 nm. The confocal images were only background corrected (subtraction of max. 10% of the maximum signal).

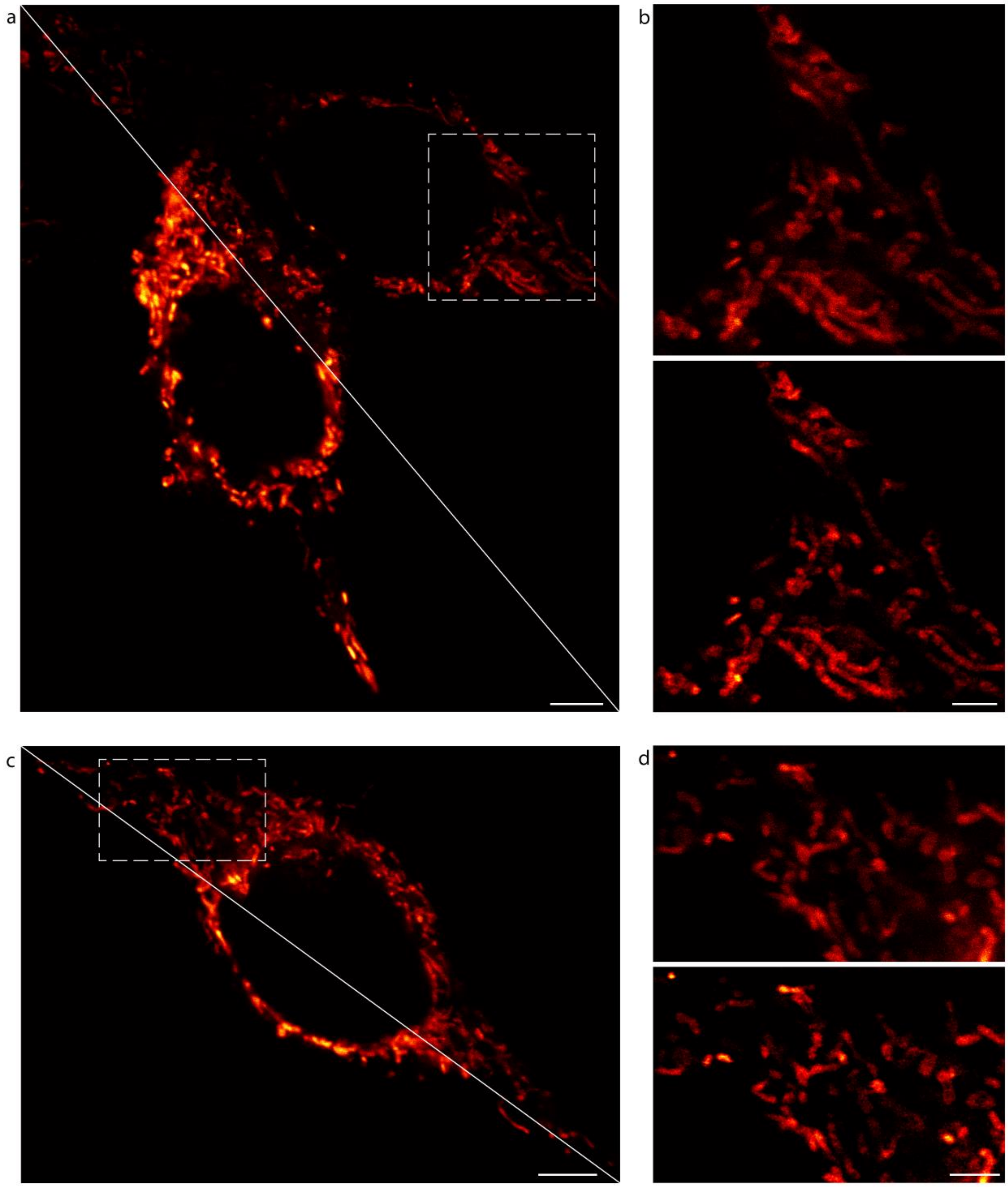

Figure S5: STED and confocal images of the mitochondrial network in living HeLa cells stained with 1 µM SiR dye **15** for 1 h. (a, c) STED images (excitation at 652 nm with 15 µW, depletion at 775 nm with 40 mW) with the corresponding confocal data (excitation at 652 nm with 7.5 µW) in the bottom left corner. All images in (a,c) are only background corrected, scale bar 5 µm. (b, d) Magnified view of the regions marked in (a, c) comparing only background corrected STED data (top) with background corrected and linearly deconvolved STED data (Lorentzian PSF) (bottom), scale bar 2 µm.

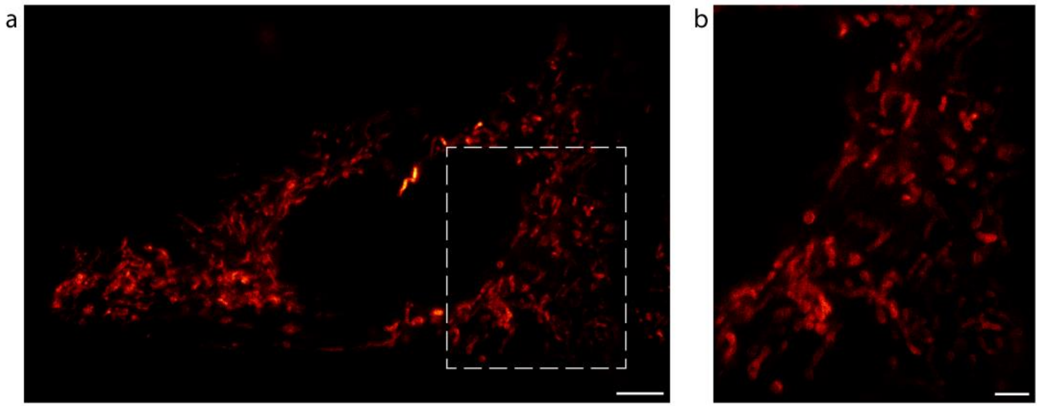

Figure S6: STED data corresponding to main text Figure 2 without deconvolution. (a) STED image (excitation at 652 nm with 15 µW, depletion at 775 nm with 40 mW) of the mitochondrial network in a living HeLa cell stained with 1 µM SiR dye **15** for 1 h, scale bar 5 µM. (b) Magnified view of the region marked in (a), scale bar 2 µm.

6. Assessment of cytotoxicity

Potential cytotoxicity of SiR dye **15** was assessed via analyzing duration and frequency of cell division using holographic time-lapse imaging with a HoloMonitor® M4 cytometer. One day prior to the experiment, U2OS (human bone osteosarcoma epithelial) cells were seeded in a 24-well cell culture plate. The medium of one half of the wells was replaced with medium containing 1 µM SiR dye **15** and the other half with fresh medium without dye. Incubation was done for 1 h at 37 °C and 5% CO_2. All wells were washed three times with medium before holographic time-lapse imaging was started using PHI HoloLids™ to ensure optimal image quality. Images were recorded for 14.5 h with one image of each well every 30 min. Data analysis was done with the HstudioM4 Software including cell segmentation, tracking of dividing cells (Figure S7), cell counting and confluency measurement (Figure S8).

Exemplary movies are available as Supplementary Material:

U2OS control Movie S2.avi and **U2OS control Movie S3.avi**: Two independent control experiments assessing undisturbed cell division of U2Os cells.

U2OS 1uM SiR dye 15 Movie S4.avi and **U2OS 1uM SiR dye 15 Movie S5.avi**: Two independent experiments assessing cell division of U2OS cells after treatment with 1 µM SiR dye **15**.

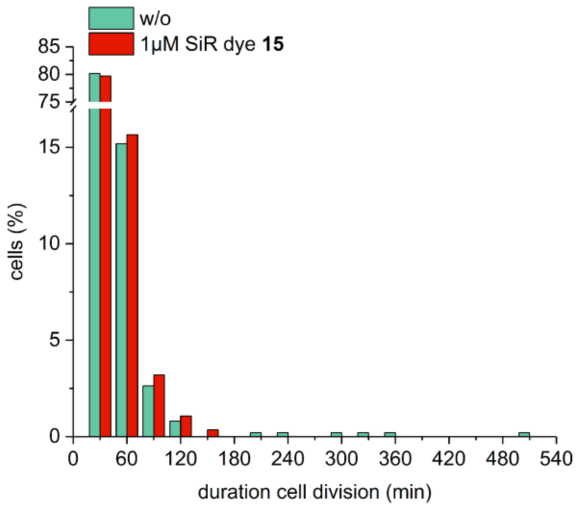

Figure S7: Comparison of the duration of cell division between untreated U2OS cells (cyan, analyzed division *N* = 494) and U2OS cells treated with 1 µM SiR dye **15** for 1 h (red, analyzed division *N* = 281). The results confirm the absence of cytotoxicity during the experiment.

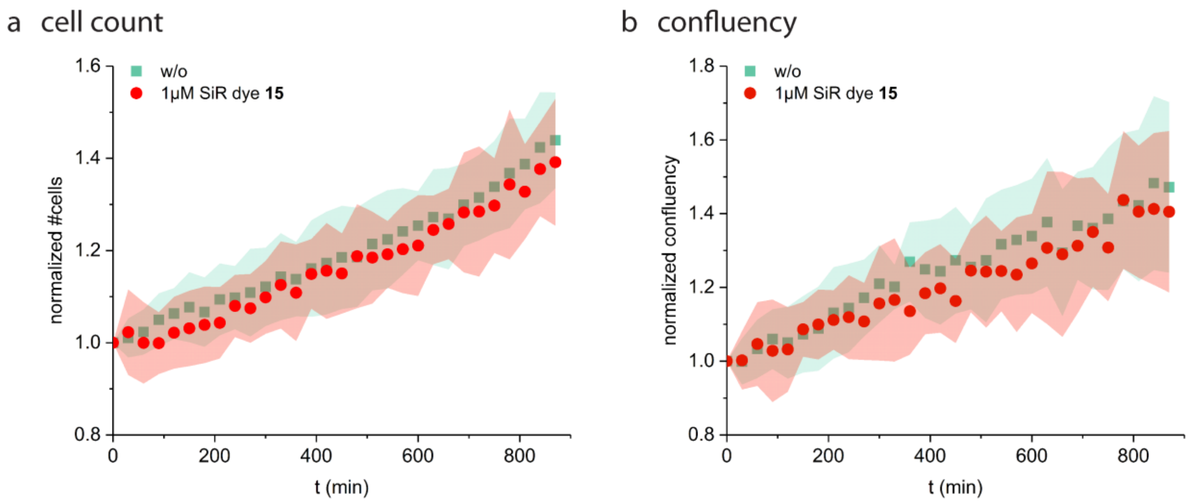

Figure S8: Comparison of the cell count (a) and the confluency (b) between untreated U2OS cells (cyan) and U2OS cells treated with 1 µM SiR dye **15** for 1 h (red). Both parameter were normalized onto the first frame (t = 0 min) and averaged (untreated cells: N = 11 experiments; treated cells: N = 8 experiments). Standard deviations were calculated and are shown as shadows in the corresponding color. P-values show no significant difference between treated and untreated cells and confirm the absence of cytotoxicity during the experiment ((a) average p = 0.521 ± 0.204, minimal p = 0.113; (b) average p = 0.574 ± 0.262, minimal p = 0.026).
